# Association of BMI with erectile dysfunction: A cross-sectional study of men from an andrology clinic

**DOI:** 10.3389/fendo.2023.1135024

**Published:** 2023-03-30

**Authors:** Yixun Liu, Xuechun Hu, Mengneng Xiong, Jiyan Li, Xiaohua Jiang, Yangyang Wan, Shun Bai, Xiansheng Zhang

**Affiliations:** ^1^ Department of Urology, Anhui Province Key Laboratory of Genitourinary Diseases, The Institute of Urology, The First Affiliated Hospital of Anhui Medical University, Anhui Medical University, Hefei, Anhui, China; ^2^ Department of Urology, The First Affiliated Hospital of University of Science and Technology of China (USTC), Division of Life Sciences and Medicine, University of Science and Technology of China, Hefei, Anhui, China; ^3^ Center for Reproductive Medicine, Renmin Hospital of Wuhan University, Wuhan, Hubei, China; ^4^ Assisted Reproduction Laboratory, Jingdezhen Maternal and Child Health Hospital, Jingdezhen, Jiangxi, China; ^5^ Reproductive and Genetic Hospital, The First Affiliated Hospital of University of Science and Technology of China (USTC), Division of Life Sciences and Medicine, University of Science and Technology of China, Hefei, Anhui, China

**Keywords:** BMI, erectile dysfunction, obesity, IIEF-5, China

## Abstract

Abnormal body mass index (BMI) is associated with an increased risk of erectile dysfunction (ED). However, the relationship between different BMI categories and the levels of ED severity remains unclear. In the current study, 878 men from the andrology clinic in Central China were recruited. Erectile function was assessed by the International Index of Erectile Function (IIEF) scores. Questionnaires included questions about demographic characteristics (age, height, weight, educational status), lifestyle habits (drinking, smoking, sleep time), and medical history. Logistic regression was used to examine the association between ED risk and BMI. The incidence of ED was 53.1%. BMI was significantly higher in men from the ED group than in those from the non-ED group (*P* = 0.01). Compared with the normal weight group, obese men had a higher risk of ED (OR = 1.97, 95% CI = 1.25-3.14, *P* = 0.004), even after adjustment for potential confounders (OR = 1.78, 95% CI = 1.10-2.90, *P* = 0.02). Moreover, the positive correlation between obesity and moderate/severe ED severity was confirmed by logistic regression analysis (moderate/severe ED, OR = 2.71, 95% CI = 1.44-5.04, *P* = 0.002), even after adjusting for potential confounders (OR = 2.51 95% CI = 1.24-5.09, *P* = 0.01). Collectively, our findings indicate a positive correlation between obesity and the risk of moderate/severe ED. Clinicians could pay more attention to moderate/severe ED patients to maintain a healthy body weight to improve erectile function.

## Introduction

Erectile dysfunction (ED), also known as impotence, is defined as the persistent inability to attain and maintain a penile erection sufficient for satisfactory sexual intercourse (1). A previous study by the Massachusetts Male Aging Study (MMAS) reported that ED is the most common sexual health issue that affected over 150 million men worldwide in 1995 and will reach over 300 million in 2025 (2). ED is associated with substantial adverse effects on physical and psychological health (3). Moreover, ED is also an independent predictive factor for cardiovascular disease and male infertility (4, 5).

Overweight/obesity, defined as abnormal or excessive fat accumulation, causes impaired quality of life. With its rapidly increasing prevalence, obesity has become a global public health concern. Sexual dysfunction, especially ED, is common among obese males. Bacon et al. studied the risk of ED in overweight/obese males and reported that obesity increases the risk of ED by approximately 1.3 times (6). Further study by Fillo et al. showed that the incidence rate of ED was directly attributed to the growing prevalence of obesity (7). Recently, a regional study found that the prevalence of ED was 42.1% and presented increasing BMI and waist circumference in young nondiabetic obese men (8). Among obese middle-aged men, 15% weight loss increased the mean International Index of Erectile Function (IIEF) score (9). In addition to weight loss by lifestyle changes, bariatric surgery has been reported to decrease ED among patients with obesity (10).

To date, several studies have shown that an elevated body mass index (BMI) is associated with an increased risk of ED among men from Europe and North America (11, 12). However, in China, whether overweight/obesity is associated with ED remains unclear. The aim of this study was to investigate the association of BMI with erectile dysfunction in young men from Central China.

## Methods

### Patients

From May 2021 to July 2022, a total of 878 men from the andrology clinic at the First Affiliated Hospital of University of Science and Technology of China (USTC) were recruited for this cross-sectional study. The exclusion criteria were as follows: (1) incomplete data for individual information; (2) incomplete data for the IIEF-5 score; and (3) unmarried. This study was approved by The First Affiliated Hospital of USTC Ethical Committee (2021-RE-064).

### Questionnaires

Questionnaires included questions about demographic characteristics (age, height, weight, educational status), lifestyle habits (drinking, smoking, sleep time), and medical history.

### Erectile function

For all participants, erectile function was assessed by the International Index of Erectile Function-5 (IIEF-5) questionnaire, which is a sensitive and specific measure for ED. The IIEF-5 questionnaire contains 5 questions on a 5-point scale. A score below 21 is considered ED, and the degree of ED is divided into three classes: severe (5–11), moderate (12–16), and mild (17–21).

### Statistical analysis

BMI was calculated as weight in kilograms divided by height in meters squared. Qualitative variables are reported as frequencies, and quantitative variables are reported as the mean ± standard deviation (SD) or as the median (interquartile range, IQR). For comparisons between the two groups, Student’s t test was used for parametric data, and Pearson’s chi-squared test (for trend) was used when needed. For comparison of more than two groups, Pearson’s chi-squared test and chi-squared test for trend were used for normal and ordinal categorical variables, respectively. Logistic regression was used to examine the association between ED risk and BMI. Covariates initially included factors possibly associated with ED risk, including age, smoking, drinking, education, chronic diseases, sex frequency, urogenital infections, varicocele and prepuce length. All tests were 2-sided using GraphPad Prism 9.0 (GraphPad Software, CA, USA), and *P* < 0.05 was considered to indicate statistical significance.

## Results

The clinical characteristics of the recruited men are shown in **Table 1**. Among 878 participants, the mean age and BMI were 31.5 years and 24.7 kg/m^2^, respectively. According to the IIEF-5 score, all subjects were divided into two groups: the ED group (IIEF-5 score > 21, 466 subjects) and the non-ED group (IIEF-5 score ≤ 21, 412 subjects). The age did not differ between the two groups (*P* = 0.35), but BMI was significantly higher in men from the ED group than in those from the non-ED group (*P* = 0.01). Analysis of four BMI categories (underweight, normal weight, overweight and obese), we found that obese men were more likely to be in the ED group than in the non-ED group (15.0% vs 8.2%, *P* = 0.02). Educational state and sex frequency were more likely to be lower in the ED group (*P* = 0.003 and *P* = 0.001, respectively). No significant differences were found in lifestyle factors (smoking and drinking) or medical history (chronic diseases, urogenital infections, varicocele and the length of prepuce).

**Table 1:** Characteristics and descriptive statistics of the whole cohort.

Clinical characteristics	Total (n=878)	ED (n=466)	Non-ED (n=412)	*P*
Age (years), mean ± SD	31.5 ± 4.9	31.6 ± 4.9	31.3 ± 4.8	0.35
BMI (kg/m^2^), mean ± SD	24.7 ± 3.5	24.9 ± 3.7	24.4 ± 3.1	0.01
< 18.5 (underweight)	30 (3.4)	17 (3.6)	13 (3.2)	0.02
18.5-23.9 (normal weight)	360 (41.0)	184 (39.5)	176 (42.7)	
24.0-28.9 (overweight)	384 (43.7)	195 (41.8)	189 (45.9)	
≥ 29 (obese)	104 (11.9)	70 (15.0)	34 (8.2)	
Smoking, n (%)				0.95
Nonsmoker	504 (57.4)	267 (57.3)	237 (57.5)	
Smoker	374 (42.6)	199 (42.7)	175 (42.5)	
Drinking, n (%)				0.93
Nondrinker	412 (46.9)	218 (46.8)	194 (47.1)	
Drinker	466 (53.1)	248 (53.2)	218 (52.9)	
Education, n (%)				0.003
Primary school	12 (1.3)	5 (1.1)	7 (1.7)	
Junior high school	148 (16.9)	99 (21.3)	49 (11.9)	
High school	148 (16.9)	78 (16.7)	70 (17.0)	
College/University	570 (64.9)	284 (60.9)	286 (69.4)	
Chronic diseases				0.11
No	788 (89.7)	411 (88.2)	377 (91.5)	
Yes	90 (10.3)	55 (11.8)	35 (8.5)	
Sex frequency (weekly), n (%)				0.001
< 1	226 (25.7)	148 (31.7)	78 (18.9)	
1-2	536 (61.1)	258 (55.4)	278 (67.5)	
> 2	116 (13.2)	60 (12.9)	56 (13.6)	
Urogenital infections, n (%)				0.31
No	774 (88.2)	406 (87.1)	368 (89.3)	
Yes	104 (11.8)	60 (12.9)	44 (10.7)	
Varicocele, n (%)				0.70
No	813 (92.6)	430 (92.3)	383 (93.0)	
Yes	65 (7.4)	36 (7.7)	29 (7.0)	
Prepuce length, n (%)				0.32
I	446 (50.8)	241 (51.7)	205 (49.8)	
II	174 (19.8)	93 (20.0)	81 (19.7)	
III	179 (20.4)	96 (20.6)	83 (20.1)	
IV	79 (9.0)	36 (7.7)	43 (10.4)	
IIEF−5 score (Median, IQR)	21 (19-23)	19 (16-20)	23 (22-24)	< 0.001

ED, erectile dysfunction; BMI, body mass index. P values were derived from Pearson’s chi-square test and Student’s t test. Prepuce length was classified into four levels according to Zhao et al (13).

The prevalence of ED according to sex frequency and BMI category is shown in **Table 2**. Among all participants, the prevalence of men with sex inactivity (less than one time per week) was 65.5%, which was higher than that of men with a sex frequency of more than one time per week (**Table 2**). Notably, among men with different BMI categories, the fraction of ED patients with sexual inactivity was higher than that of sexually active participants, although the significant difference was only in men with normal weight and overweight (**Table S1**). In addition, the prevalence of ED in our study was 53.1%, with obese men having a higher prevalence than those with a normal BMI (67.3% vs 51.1%) (**Table 2**).

**Table 2:** Prevalence of ED according to sex frequency and BMI category.

	Participants (n=878)		
	N	Prevalence (%)	95% CI (%)
Sex frequency (weekly)			
< 1	226	65.5	59.1-71.4
1-2	536	48.1	43.9-52.4
> 2	116	51.7	42.7-60.6
BMI (kg/m^2^)			
< 18.5 (underweight)	30	56.7	39.2-72.6
18.5-23.9 (normal weight)	360	51.1	46.0-56.2
24.0-28.9 (overweight)	384	50.8	45.8-55.7
≥ 29 (obese)	104	67.3	57.8-75.6
Overall	878	53.1	49.8-56.4

BMI, body mass index; CI, confidence interval; ED, erectile dysfunction.

To analyze the association between ED and clinical characteristics, we performed a logistic regression analysis model, and the results are shown in **Table 3**. Participants with a low education level (junior high school) had a higher risk of ED (OR = 2.04, 95% CI = 1.40-2.99, *P* = 0.002). In addition, the frequency of sex less than once a week was significantly associated with the risk of ED (OR = 1.77, 95% CI = 1.12-2.80, *P* = 0.01). Compared with the normal weight group, obese men had a higher risk of ED (OR = 1.97, 95% CI = 1.25-3.14, *P* = 0.004), even after adjustment for potential confounders (OR = 1.78, 95% CI = 1.10-2.90, *P* = 0.02).

**Table 3:** Logistic regression analyses for ED and BMI, education and sex frequency.

	Crude		Adjusted	
	OR (95% CI)	*P*	OR (95% CI)	*P*
Age	1.01 (0.98-1.04)	0.35		
Smoking	1.01 (0.77-1.32)	0.95		
Drinking	1.01 (0.78-1.32)	0.93		
Education				
Primary school	0.72 (0.21-2.28)	0.58		
Junior high school	2.04 (1.40-2.99)	0.0002		
High school	1.12 (0.78-1.61)	0.53		
College/University	Ref			
Chronic diseases	1.44 (0.93-2.27)	0.11		
Sex frequency				
< 1	1.77 (1.12-2.80)	0.01		
1-2	0.87 (0.58-1.29)	0.48		
> 2	Ref			
Urogenital infections	1.24 (0.82-1.88)	0.32		
Varicocele	1.11 (0.67-1.85)	0.70		
Prepuce length				
I	Ref			
II	0.98 (0.69-1.39)	0.90		
III	0.98 (0.70-1.39)	0.93		
IV	0.71 (0.44-1.15)	0.17		
BMI	1.05 (1.01-1.10)	0.01		
< 18.5 (underweight)	1.25 (0.59-2.70)	0.56	1.18 (0.54-2.62)	0.68
18.5-23.9 (normal weight)	Ref		Ref	
24.0-28.9 (overweight)	0.99 (0.74-1.32)	0.93	1.00 (0.74-1.34)	0.98
≥ 29 (obese)	1.97 (1.25-3.14)	0.004	1.76 (1.09-2.86)	0.02

BMI, body mass index; ED, erectile dysfunction. ED-adjusted model, adjusted for age, smoking, drinking, education, chronic diseases, sex frequency, urogenital infections, varicocele and prepuce length.

In terms of all participants, a significantly higher frequency of obesity was found in mild (3.8%), moderate (18.3%) and severe ED (45.2%) in comparison with men with normal weight (*P* = 0.001) (**Table 4**).

**Table 4:** Frequency distribution of BMI according to IIEF-5 score.

	Total (n=878)	< 18.5 (Q1, n=30)	18.5-23.9 (Q2, n=360)	24.0-28.9 (Q3, n=384)	≥ 29 (Q4, n=104)	*^a^P*	*^b^P*	*^c^P*
IIEF−5 score, n (%)						0.57	0.94	0.001
5-11	20 (2.3)	1 (3.4)	7 (1.9)	8 (2.1)	4 (3.8)			
12-16	97 (11.1)	3 (10.0)	37 (10.3)	38 (9.9)	19 (18.3)			
17-21	349 (39.7)	13 (43.3)	140 (38.9)	149 (38.8)	47 (45.2)			
22-25	412 (46.9)	13 (43.3)	176 (48.9)	189 (49.2)	34 (32.7)			

BMI, body mass index; IIEF-5, International Index of Erectile Function questionnaire. Chi-square test for trend.

a P, Q2 vs Q1;

b P, Q2 vs Q3;

c P, Q2 vs Q4.

The positive correlation between obesity and ED severity (mild and moderate) was confirmed by logistic regression analysis (moderate/severe ED, OR = 2.71, 95% CI = 1.44-5.04, *P* = 0.002; mild ED, OR = 1.74, 95% CI = 1.06-2.86, *P* = 0.03) (**Table 5**). Furthermore, after adjusting for potential confounders, a high risk of moderate/severe ED was still observed in obese men (OR = 2.51 95% CI = 1.24-5.09, *P* = 0.01).

**Table 5:** Logistic regression for the association between BMI and ED severity.

	IIEF-5 score				
	5-16		17-21		22-25
	OR (95% CI)	*P*	OR (95% CI)	*P*	
Crude					
< 18.5	1.23 (0.33-3.68)	0.73	1.26 (0.56-2.82)	0.58	Ref
18.5-23.9	Ref		Ref		Ref
24.0-28.9	0.97 (0.61-1.55)	0.91	0.99 (0.73-1.35)	0.95	Ref
≥ 29	2.71 (1.44-5.04)	0.002	1.74 (1.06-2.86)	0.03	Ref
Adjusted					
< 18.5	1.01 (0.26-3.30)	0.98	1.15 (0.50-2.68)	0.74	Ref
18.5-23.9	Ref		Ref		Ref
24.0-28.9	0.95 (0.58-1.56)	0.85	0.99 (0.72-1.36)	0.94	Ref
≥ 29	2.51 (1.24-5.09)	0.01	1.48 (0.89-2.50)	0.13	Ref

BMI, body mass index; ED, erectile dysfunction. ED-adjusted model, adjusted for age, smoking, drinking, education, chronic diseases, sex frequency, urogenital infections, varicocele and prepuce length.

## Discussion

ED is a common sexual disorder and is a concern as a factor of metabolic syndrome in men (14, 15). This observational study investigated the relationship between BMI and ED risk and found that participants with obesity had a 1.3-fold greater risk of ED than participants with normal weight. After adjusting for potential confounders, obese men had an increased risk of moderate-severe ED.

The guidelines of the European Association of Urology (EAU) show a high incidence and prevalence of ED worldwide (16). In China, the prevalence of ED is approximately 20%-40% in men below 50 years of age (17). In the present study, the prevalence of ED was 53.1%, with a mean age of 31.5 years, and more than 99% were younger than 50 years. The frequency of sexual activity in approximately one-third of the ED patients was less than one time a week, which was significantly higher than the frequency in non-ED patients (19%), indicating that sexual inactivity was more common in ED patients. Notably, a subset of subjects were men who underwent fertility evaluation. Since sexual dysfunction, including ED, leads to impaired fertility through natural conception in men of reproductive age, the ED prevalence in our study population is relatively high, although sexual dysfunction accounted for only 0.4-4.6% of male infertility (5). These results suggest that clinical screening for the risk of ED should include the assessment of male reproductive health.

We observed a U-shaped relationship between BMI and ED prevalence, with a higher prevalence in underweight and obese participants than in normal and overweight participants. The results were similar to those of a population-based study by Cheng et al. but inconsistent with those of the Health Professionals Follow-Up Study (HPFS, a prospective cohort study) (18, 19). Analysis of BMI categories used in these studies revealed that both Cheng and our studies divided the underweight (BMI < 18.5 kg/m^2^) subgroup, while the HPFS study used a BMI less than 23 kg/m^2^ as the reference group. It is possible that different BMI cutoffs in European and Asian populations classified by the WHO result in different BMI categories being used (20). As a result, the decreasing range of the U-shaped relationship between BMI and ED was not present in the HPFS study. Nevertheless, the present study extended the high prevalence of ED in underweight and obese men.

Evidence from animals to humans has reported that obesity plays a key role in male sexual dysfunction, including premature ejaculation and ED, although ED is mainly considered an age-related disease (21, 22). It has been demonstrated not only that obesity is a strong and independent risk factor for ED but also that ED risk is positively correlated with the level of obesity (7). A recent study showed that the prevalence of ED was 42% in nondiabetic young obese men from a primary care-based cohort (8). Moreover, a meta-analysis including three studies demonstrated that the prevalence of ED in obese men with a BMI > 30 kg/m^2^ was approximately 71-73% (21), which was similar to the value (67%) obtained from men with BMI > 29 kg/m^2^ in the current study. The prevalence of ED is approximately 1.3 times higher for obese men than for normal weight men, confirming the relationship between obesity and the incidence of ED.

Although obesity is a well-documented risk factor for ED, the association between obesity and the levels of ED severity remains unclear. Our results show that the prevalence of severe ED in underweight and obese men was higher than that in those who were normal weight. Notably, overweight (BMI = 24.0-28.9 kg/m^2^) and normal weight men had similar incidence rates of mild, moderate and severe ED, indicating that the prevalence of ED was not increased even though they were overweight. Considering that the mean BMI (24.7 kg/m^2^) reached the overweight range, we assumed that the observations of our study could be partly explained by the enrolled populations.

Our data also revealed a positive correlation of obesity with moderate/severe ED risk after adjusting for confounders. According to the role of obesity in metabolic syndrome, the high risk of moderate/severe ED may be due to pathophysiologic processes, including oxidative stress, inflammation, and insulin and leptin resistance. This might partly explain the elevated prevalence of chronic diseases (hypertension, hyperlipidemia, and diabetes) in the ED groups (ED: 11.8%, non-ED: 8.5%) in the current study. Moreover, the efficacy of phosphodiesterase type 5 inhibitors (PDE5-i) on erectile function is reduced in obese men (23). Obesity evaluation can be a useful biomarker in those with severe ED and PDE5-i nonresponders. However, unlike obesity, both crude and adjusted analyses showed that underweight was not associated with ED risk. The relatively small number of underweight men enrolled may limit the statistical power.

It is important to note that a randomized controlled trial was performed to evaluate weight reduction on ED in obese men (9). The results showed that lifestyle changes are associated with improvements in erectile function. In addition, weight loss by bariatric surgery has been reported to improve erectile function in obese men (10). Accordingly, it seems to be reversible that weight loss from exercise, diet, or surgery could reverse ED and provide a view of the cost-effectiveness for the treatment of ED.

The current study has several strengths. First, the mean age of the recruited men was 31.5 years, reducing the effect of age on ED risk. Second, several potential confounders, including age, BMI, lifestyle styles (smoking and drinking), educational status and medical history (chronic diseases, urogenital infections, varicocele and the length of prepuce), were included in our study, which gives more statistical power. Third, classification of the BMI categories was based on the recommendations of WHO Asian BMI cutoffs. Fourth, the relationship between BMI categories and the severity of ED according to the IIEF-5 was assessed.

We also acknowledge that this study has several limitations. First, our study was limited to an association based on the cross-sectional study design. Second, only healthy or subfertile men who underwent infertility investigations were selected, and further studies across other populations will be needed. Third, metabolic parameters and penile duplex ultrasound were not measured in the present study. Finally, recall bias may have occurred in this study when data were obtained from questionnaires.

In conclusion, the findings in this study indicate that obesity is associated with an increased risk of moderate/severe ED. Given the high prevalence of ED in obese men, clinicians could pay more attention to moderate/severe ED patients to maintain a healthy body weight to improve erectile function.

## Data availability statement

The original contributions presented in the study are included in the article/**Supplementary Material**. Further inquiries can be directed to the corresponding author.

## Ethics statement

This study was approved by The First Affiliated Hospital of USTC Ethical Committee (2021-RE-064). The patients/participants provided their written informed consent to participate in this study.

## Author contributions

YL, SB, and XZ designed the research study. XH, MX, JL, XJ, YW, and SB contributed to the data acquisition. YL, XH, SB, and XZ analyzed the data. YL and SB wrote the paper. All authors contributed to the article and approved the submitted version.
