# Supplementary material for: Association of BMI with erectile dysfunction: A cross-sectional study of men from an andrology clinic
Source: Front Endocrinol (Lausanne). 2023 Mar 30;14:1135024. doi: 10.3389/fendo.2023.1135024 (PMC10101565; doi:10.3389/fendo.2023.1135024)
Supplement: Supplementary file 1 [file Table_1.docx]

Table S1. Frequency distribution of ED according to sex frequency among men with different BMI categories

| Sex frequency (weekly), n (%) | < 18.5 | | 18.5-23.9 | | 24.0-28.9 | | ≥ 29 | |
| --- | --- | --- | --- | --- | --- | --- | --- | --- |
|  | ED  (n=17) | Non-ED  (n=13) | ED  (n=184) | Non-ED  (n=176) | ED  (n=195) | Non-ED  (n=189) | ED  (n=70) | Non-ED  (n=34) |
| < 1 | 4 (23.5) | 2 (15.4) | 58 (31.5) | 32 (18.2) | 57 (29.2) | 36 (19.0) | 29 (41.4) | 8 (23.5) |
| ≥1 | 13 (76.5) | 11 (84.6) | 126 (68.5) | 144 (81.8) | 138 (70.8) | 153 (81.0) | 41 (58.6) | 26 (76.4) |
|  | *P* = 0.58 | | *P* = 0.004 | | *P* = 0.02 | | *P* = 0.07 | |

BMI: body mass index. ED: erectile dysfunction.
